# Quantitative EEG Tomography of Early Childhood Malnutrition

**DOI:** 10.3389/fnins.2018.00595

**Published:** 2018-08-28

**Authors:** Alberto Taboada-Crispi, Maria L. Bringas-Vega, Jorge Bosch-Bayard, Lidice Galán-García, Cyralene Bryce, Arielle G. Rabinowitz, Leslie S. Prichep, Robert Isenhart, Ana Calzada-Reyes, Trinidad VIrues-Alba, Yanbo Guo, Janina R. Galler, Pedro A. Valdés-Sosa

**Affiliations:** ^1^The Clinical Hospital of Chengdu Brain Science Institute, MOE Key Lab for Neuroinformation, University of Electronic Science and Technology of China, Chengdu, China; ^2^Informatics Research Center, Universidad Central Marta Abreu de las Villas, Santa Clara, Cuba; ^3^Cuban Neuroscience Center, Havana, Cuba; ^4^Institute for Neurobiology, Universidad Nacional Autonoma de Mexico, Juriquilla, Mexico; ^5^Barbados Nutrition Study, Bridgetown, Barbados; ^6^Department of Psychiatry, School of Medicine, New York University, New York, NY, United States; ^7^Newport Brain Research Laboratory, Newport Beach, CA, United States; ^8^Chester M. Pierce MD Division of Global Psychiatry, Massachusetts General Hospital, Boston, MA, United States; ^9^Center on the Developing Child, Harvard University, Cambridge, MA, United States

**Keywords:** EEG, qEEG, quantitative EEG, tomography qEEGt, protein energy malnutrition PEM, neurodevelopment

## Abstract

The goal of this study is to identify the quantitative electroencephalographic (qEEG) signature of early childhood malnutrition [protein-energy malnutrition (PEM)]. To this end, archival digital EEG recordings of 108 participants in the Barbados Nutrition Study (BNS) were recovered and cleaned of artifacts (46 children who suffered an episode of PEM limited to the first year of life) and 62 healthy controls). The participants of the still ongoing BNS were initially enrolled in 1973, and EEGs for both groups were recorded in 1977–1978 (at 5–11 years). Scalp and source EEG Z-spectra (to correct for age effects) were obtained by comparison with the normative Cuban Human Brain Mapping database. Differences between both groups in the z spectra (for all electrode locations and frequency bins) were assessed by *t*-tests with thresholds corrected for multiple comparisons by permutation tests. Four clusters of differences were found: (a) increased theta activity (3.91–5.86 Hz) in electrodes T4, O2, Pz and in the sources of the supplementary motor area (SMA); b) decreased alpha1 (8.59–8.98 Hz) in Fronto-central electrodes and sources of widespread bilateral prefrontal are; (c) increased alpha2 (11.33–12.50 Hz) in Temporo-parietal electrodes as well as in sources in Central-parietal areas of the right hemisphere; and (d) increased beta (13.67–18.36 Hz), in T4, T5 and P4 electrodes and decreased in the sources of bilateral occipital-temporal areas. Multivariate Item Response Theory of EEGs scored visually by experts revealed a neurophysiological latent variable which indicated excessive paroxysmal and focal abnormality activity in the PEM group. A robust biomarker construction procedure based on elastic-net regressions and 1000-cross-validations was used to: (i) select stable variables and (ii) calculate the area under ROC curves (AUC). Thus, qEEG differentiate between the two nutrition groups (PEM vs Control) performing as well as visual inspection of the EEG scored by experts (AUC = 0.83). Since PEM is a global public health problem with lifelong neurodevelopmental consequences, our finding of consistent differences between PEM and controls, both in qualitative and quantitative EEG analysis, suggest that this technology may be a source of scalable and affordable biomarkers for assessing the long-term brain impact of early PEM.

## Introduction^1^

Malnutrition remains a persistent problem worldwide that affects up to 22.9% of children in developing countries (UNICEF et al., 2017). Protein-energy malnutrition (PEM), is defined as insufficient protein consumption and/or caloric intake and is the most prevalent form of malnutrition, affecting an estimated 155 million children globally—even in industrialized nations such as the United States (Global Health Observatory [GHO], 2016). Nearly half of all deaths of children under the age of 5 is attributable to undernutrition (UNICEF et al., 2017). An increasing number of malnourished children now survive to adulthood thanks to improved public health measures. However, since PEM can have long-term effects on cognition and behavior (Grantham-McGregor et al., 2007) as well as on brain development, this has created a new social problem (Bakken, 2016). Early PEM exposure during a critical period of brain development (from the second trimester of pregnancy to age 2), has been shown to produce permanent brain related changes, whereas exposure at later ages is considered reversible (Dickerson et al., 1967). Thus, timely, population-based identification of children at risk for developmental problems after early PEM could be the basis for global personalized intervention programs.

Although advanced neuroimaging techniques such as MRI or PET may be used to identify a neural signature of early PEM, they are costly and have the disadvantage of low throughput. Consequently, these techniques are not feasible for the development of scalable screening programs in any country, much less in developing countries where PEM is most prevalent. Quantitative electroencephalography (qEEG) is an alternative technology that can be utilized to determine biomarkers suitable for global health applications. qEEG is currently one of the most non-invasive, inexpensive, efficient and useful techniques for assessing brain development and dysfunction. qEEG is based on analysis of the resting state EEG power spectrum (Hernandez-Gonzalez et al., 2011) which is either averaged over broad frequency bands (John et al., 1977) or in narrower bins (Szava et al., 1994) to yield a set of features. These features are then transformed toward Gaussianity and converted to *z* scores, based on age dependent means and standard deviations. qEEG thus provides the topographic distribution of EEG spectra abnormality at the scalp. An important advancement in this methodology is tomographic qEEG (qEEGt), which utilize features derived from the spectra of cortical current source densities, rather than those derived from the surface EEG electrodes. The surface EEG is the reflection on the scalp, via volume conduction, of deep sources which are located in actual brain structures. Bosch-Bayard et al. (2001) qEEGt has been validated in population based multimodal neuroimaging studies (Hernandez-Gonzalez et al., 2011) and has been shown to be more accurate than qEEG in detecting brain dysfunction in newborns during quiet sleep, as determined by the area under the receiving operating curves (ROC) (Bosch-Bayard et al., 2012). There are currently no published studies that have fully examined either qEEG or qEEGt in malnourished children. However, there are several earlier studies using visual inspection of the EEG (Stoch and Smythe, 1967; Baraitser and Evans, 1969) which found marked differences (e.g., slower theta, less alpha) in malnourished children compared to controls. These EEG patterns were later confirmed by computerized studies of the EEG spectrum (Bartel et al., 1979; Robinson et al., 1995), using different methodologies (quiet sleep and photic stimulation versus awake condition, no stimulation). Note that these results are for scalp computerized EEG results only. Therefore, in this paper we also calculate scalp measures for comparison with these prior studies.

In the current study, we have carried out a complete qEEG/qEEGt analysis to document the long-term effects of early childhood PEM on brain function. We used archival EEG data collected in childhood, at ages 5–11 years, derived from the Barbados Nutrition Study (BNS), a longitudinal study that has followed individuals who suffered from moderate-severe PEM limited to the first year of life, as well as a rigorously selected matched set of healthy controls, for nearly 50 years (Ramsey, 1979). Both groups have been followed over the life span and their offspring have also been studied. Significant problems in cognitive and behavioral function, soft neurological signs and health outcomes have been documented in childhood, adolescence and middle adulthood following a history of PEM (Peter et al., 2016; Waber et al., 2016). Using these EEG data, we have been afforded a unique opportunity to evaluate the sensitivity of both qEEG and qEEGt analysis. This will provide the basis for determining potential biomarkers of early childhood malnutrition. At the same time, we also carried out analyses of the visual inspection of the EEG to identify the predictive value of our quantitative analyses as compared with traditional visual EEG clinical evaluation.

## Materials and Methods

### Study Site

The current study was conducted in Barbados, a Caribbean country whose population is approximately 280,000 at present. The demographic makeup is 92% African/Caribbean origin, 4% Caucasian and 4% individuals of Asian, Lebanese and Syrian descent. In 1970, the infant mortality rate was 46 per 1,000 live births. Today it stands at 7.8 and Barbados is ranked as 52 on the Human Development Index (United Nations Development Programme United Nations Development Programme [UNDP], 2016). Whereas moderate-severe cases of infant malnutrition were of significant concern when this study was undertaken in the 1970’s, infant malnutrition is now virtually eliminated from the island due to its improved economy and the impact of island-wide nutrition-related education (Ramsey, 1979; Galler et al., 1984).

### Description of Sample

Participants consisted of children born between 1967 and 1972 who were diagnosed with PEM in the first year of life (*n* = 129, 52 females, 77 males) and a matched healthy control group (*n* = 129, 52 females, 77 males). Inclusion criteria were, as follows: (UNICEF et al., 2017) birth weight > 2500 g; (Global Health Observatory [GHO], 2016) Apgar score ≥ 8 at birth; (Grantham-McGregor et al., 2007) no birth complications; and (Bakken, 2016) no encephalopathic events in childhood. The PEM group experienced a single episode of Grade II or III PEM in the first year of life based on clinical diagnosis at the time of admission to the Queen Elizabeth Hospital (Galler et al., 1983a; Gomez et al., 2000). The control group were classmates of the index group who met the same inclusion criteria as the PEM group but lacked a history of malnutrition. Three healthy classmates were identified as possible matches for each index child based on age (±3 months), gender and handedness. Final selection was based on parental consent and access to birth and preschool health records. In later waves of data collection, children with histories of kwashiorkor (*n* = 54) were also included, but EEG data was not collected in this group (Galler et al., 1987a,b,c). All PEM children were enrolled in a national program (NIP- Nutrition Intervention Program)- that provided subsidized food, maternal nutrition education, regular home visits, a pre-school nursery, health monitoring and medical care from hospital discharge until 12 years of age (Ramsey, 1979), ensuring that no child had further episodes of malnutrition. The BNS participants and their children have now been followed for 45+ years and seven waves of data collection have been conducted (see **Supplementary Figure S1**). The original participants or first generation (G1), are the subjects of this report and were 5–11 years of age at the time. Written informed consent was obtained from all participants. Approval for this study was granted by the Ethics Committee of the Ministry of Health, Barbados, the Judge Baker Children’s Centre Human Research Review Committee (Assurance No. FWA 00001811) and the Massachusetts General Hospital IRB (2015P000329/MGH). Participants were compensated for their time and travel to and from the BNS research center.

### EEG Data Acquisition

**FIGURE 1:**
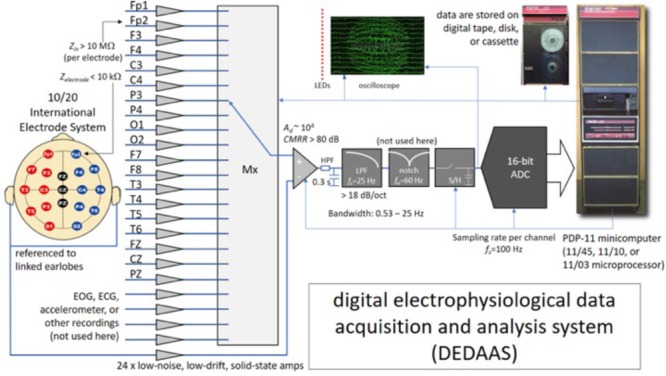
System used for the EEG data acquisition process in Barbados. *Z_in_*, input impedance of the EEG channels (per electrode); *Z_electrode_*, contact impedance between the electrode and the scalp; EOG, ECG, electrooculography, electrocardiography; LEDs, light emitting diodes; LPF, low-pass (antialiasing) filter; HPF, high-pass filter; Mx, multiplexor; ADC, analog-to-digital converter; S/H, sample and hold; CMRR, common mode rejection ratio; *A_d_*, differential gain of the channels; *f_s_*, sampling frequency; *f_c_*, cut-off frequency of the low-pass (antialiasing) filter; *f_o_*, central frequency of the notch filter.

The EEGs were recorded in 1977–1978 by trained staff at the Barbados Nutrition Centre, who were blind to the child’s nutritional history. Children were accompanied to the center by their mothers, primary caretakers or a relative, who were instructed not to administer any medication in the days before the test. A designated room was used for the EEG testing. During the experiment, the child rested on a comfortable armchair, and was given instructions to close their eyes but not to sleep. The custom-designed digital electrophysiological data acquisition and analysis system (DEDAAS) used for testing was provided by Prof. E. Roy John from NYU Brain Research Labs (Thatcher and John, 1977) (**Figure 1**).

The DEDAAS device consisted of 24 solid-state EEG amplifiers with the following characteristics: precise fixed differential gain (*A_d_* in the order of 10^4^), low noise (in the order of tens of μV), high common-mode rejection ratios (CMRR greater than 80 dB), sharp 60-Hz (notch-filter); high-input impedance (*Z_in_* higher than 10 MΩ per electrode); and an antialiasing low-pass filter (LPF) with a cut-off frequency was at 25 Hz, with a roll-off steeper than -18 dB/octave. The output of the amplifiers was fed through an 12 bit A/D converter with a sampling frequency (*f_s_*) of 100 Hz. into a PDP-11 minicomputer that both calibrated the amplifiers and checked the electrode impedance automatically. Simultaneous monopolar recordings were obtained of the 10/20 International Electrode System (Fp1, Fp2, F3, F4, C3, C4, P3, P4, O1, O2, F7, F8, T3, T4, T5, T6, FZ, CZ, and PZ), all referenced to linked earlobes. Data was stored on digital tape. Frequency and voltage limits specified for every channel were constantly monitored, permitting the computer automatically to reject data contaminated by eye or body movement, or by high electrode impedance.

### EEG Sample and Preprocessing

Two hundred and fifty eight digital resting state EEG recordings were initially collected and 137 of these were recovered by LP and RI in September 2016. The remaining records were considered to be lost, or no longer accessible due to file corruption. To facilitate the recognition of each EEG recording, they were labeled using a 7-digit code (group-serial number of the subject in the BNS-the initial letters of the surname and names). The raw EEG was divided in epochs of 256 samples.

AT-C and RI converted the raw EEG records to EEGLAB format. Two expert neurophysiologists (AC-R and TV-A) carried out visual inspection of the recovered EEG data, using tools pertaining to both time and frequency domains. Somnolence and/or different sleep stages were found in 29 children. The criteria to identify somnolence were drop-out and slowing of alpha frequency and/or sleep phase one (vertex spikes) or phase two (spindles and K complexes in central regions). These participants were excluded from this study leaving 108 usable recordings (PEM Group: 17 females and 29 males; Control Group: 28 females and 34 males).

**FIGURE 2:**
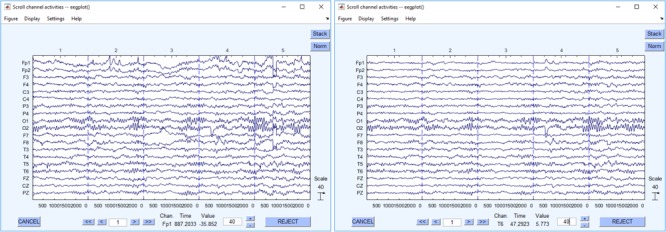
Recovered EEG recordings. The left figure shows the EEG raw data. The right one shows the same EEG data segment after cleaning.

Despite the built-in automatic artifact rejection procedures of DEDDAS, some recordings displayed ocular and muscular artifacts, mainly those in the frontal leads, Fp1 and Fp2. These were eliminated using the AAR plug-in from the EEGLAB 13.6.5b toolbox (Gomez-Herrero et al., 2006; De Clercq et al., 2006). An example of the difference between the data before and after cleaning is showed in **Figure 2**. This figure also illustrates the quality of the recovered archival EEGs.

**FIGURE 3:**
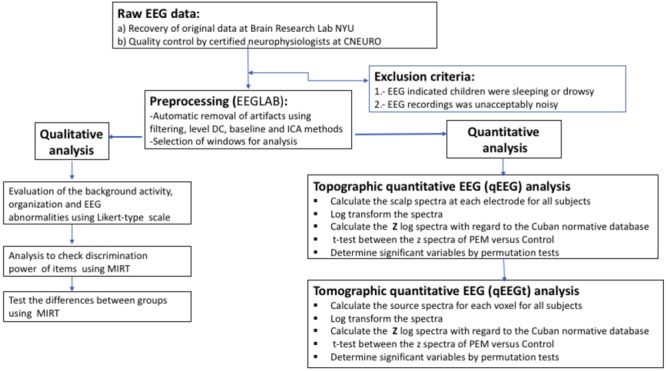
Flow of the EEG analysis. Processing steps from raw EEG to statistical results.

The overall flowchart of EEG processing is shown in **Figure 3**.

### Qualitative EEG Analysis

Data collection, evaluation and visual inspection of the EEG recordings were carried out under blind conditions, conforming to the guidelines for resting state EEG analysis from the International Federation of Clinical Neurophysiology IFCN^2^. EEG recordings were considered normal if they contained adequate organization of background activity (according to age), a well-defined spatial differentiation, rhythmic alpha activity and the absence of slow or paroxysmal wave activity. Slow EEG activity was defined as the presence of persistent non-rhythmic theta-delta slow waves. Paroxysmal activity was defined as spikes, sharp waves and polyspike-slow wave complexes. EEGs representing both types of abnormalities were included in the Slow and Paroxysmal category. We used the a global scoring scale (GTE) to quantify the group differences in the observed EEG abnormalities (Jonkman, 1989; De Weerd et al., 1990). Originally designed to evaluate the predictive power of the EEG in dementia, this scale was modified (by TV-A and AC-R) for use in the current study and adjusted for the participant age (see the modified scale in the **Supplementary Material S1**). The original scale consisted of 5 items: frequency of rhythmic background activity, diffuse slow activity, paroxysmal activity, focal abnormality, sharp wave activity, and a total score summarizing all 5 items. All EEG items were evaluated such that higher scores reflected greater abnormality. We used multivariate item response theory (MIRT) (Chalmers, 2012) to select the most informative items and identify their optimal linear combination obtained by a non-linear factor analysis to produce an overall score. This overall score reflected a latent variable which we refer to as “neurophysiological status” NS). It is known that latent variables identified by MIRT are independent of the evaluator and robust against chance fluctuations in score recording (Fox, 2010). In a preliminary analysis, each item was examined separately to assess how reliably its response alternatives were distinguished. Next, the latent variable was integrated into a mixed effects model. By using this procedure implemented in the MIRT package in R (Møller, 1986), it was possible to account for the variability inherent to each examiner by including it as a random effect when testing for differences between control and PEM groups.

### Quantitative EEG Analysis (qEEG and qEEGt)

(1) The two feature sets extracted from the BNS data were, as follows:

(a) qEEG topography, or simply qEEG, the array of EEG scalp (electrode) spectra. The scalp EEG cross-spectrum was estimated using Bartlett’s method (Mazziotta et al., 2001) by averaging the cross-periodograms of at least 20 consecutive and non-overlapping epochs of 256 samples each (i.e., 2.55 s), ignoring any discontinuity in the recovered records. This procedure yielded cross-spectra for a set of 48 frequency bins ranging from 0.78 to 19.14 Hz with a resolution of 0.39 Hz. Global differences in scale among EEG data were handled using geometric power correction (Hernández et al., 1994). For qEEG only, the diagonal elements of the cross-spectra were retained, which yielded a final set of 912 scalp spectral features (19 channels^∗^48 frequencies).
(b) tomographic qEEG or simply qEEGt, the set of EEG spectra for all 3244 sources defined in the brain cortex (excluding basal ganglia). The volumetric sources were estimated from the EEG cross-spectra by the VARETA (Variable Resolution Electromagnetic Tomography) electrophysiological source imaging method. This is a discrete spline EEG inverse solution. It is based on a forward model that incorporates anatomical constraints using the template of ICBM (probabilistic brain atlas) created by the Montreal Neurological Institute (MNI) (Mazziotta et al., 2001). The procedure yielded 155712 features (3244 sources^∗^48 frequencies)
(c) Both feature sets were log transformed toward the Gaussian distribution. (Gasser et al., 1988)

(2) Next, *z* scores were obtained by comparing qEEG in the PEM and Control participants with the Cuban normative database using age as a covariate (John et al., 1988; Galán et al., 1994; Szava et al., 1994) and qEEGt in (Hernandez-Gonzalez et al., 2011). The result feature sets are scalp z spectra for qEEG and source z spectra for qEEGt. The expression for the well known z transform is: $Z=\frac{x-\mu (age)}{\sigma (age)}$ where x is any qEEG or qEEGt parameters standardized with the age dependent mean and standard deviation.

qEEG and qEEGt methods have been summarized extensively elsewhere (Niedermeyer et al., 2004; Thatcher and Lubar, 2009; Hernandez-Gonzalez et al., 2011). They were implemented using the qEEGt MATLAB-based (Hernandez-Gonzalez et al., 2011) software available by request from the authors.

In this paper, we employed a mass univariate approach to compare qEEG and qEEGt features between both groups. A sample t test was carried out for all variables, and results were reported only for tests above a threshold corrected for multiple comparisons using permutation methodology, and specifically the non-parametric combination (NPC). (Pesarin and Salmaso, 2010) “The NPC methodology allows for straightforward extension to multiple testing and multiple comparisons…”. We utilized a combination also described by this author: “In particular, Tippett’s combining function ….can perform step-down procedures which enable computation to be speeded up….. Special cases of Tippett’s combining functions are the ‘max t test’ and the ‘max chi-square…….”

We applied the max *t*-test permutation test for qEEG described for the first time by our group (Galán et al., 1994) to evaluate the differences between groups using the z spectra of both topographic (qEEG) and tomographic (qEEGt) features (see also Blair and Karniski, 1993), which is currently a standard in neuroimaging research (Winkler et al., 2016).

Permutation tests have the following advantages: the tests are distribution-free and provide a *p*-value for the test statistic that has the correct Type I error rate, regardless of the number of univariate tests for combined assessment. In addition, no assumptions of an underlying correlation structure are required, and they provide exact *p*-values for any number of subjects, frequency points and recording sites. The correction of the *p*-values is converted into a global statistic that contrasts the null hypothesis using the non-parametric combination of the *p*-values.

Thus, the *t* statistics were calculated for all derivations and frequencies (912 variables) or for all sources and frequencies (3244 variables). The *max t* statistic was calculated in each case, where *max t* represents the maximum of the *t* statistic over all variables considered and is used to summarize differences between two Z spectrums. The algorithm for the permutation test consisted of:

(1) Permutation of the observations between the groups repeatedly for all variables. In each repetition both statistics were calculated for the 912 variables at electrode level (19 electrodes by 48 frequencies) as well as for the 155,712 variables of the source analysis (3244 sources by 48 frequencies). Each test was carried out using 10000 random permutations.
(2) The empirical distributions of the *t* and max *t* statistic were calculated.
(3) The 95th percentiles were obtained for each statistic using the empirical distributions of the above step. This value was used as the threshold of significance.
(4) This permutation procedure allowed us to report only those *t*-tests that were significant at the global *p* < 0.05 level (corrected for multiple comparisons). In an earlier paper (Bosch-Bayard et al., 2012), we analyzed the power of qEEGt procedures regarding the size sample. Power calculations from that report (shown in **Figure 2** of the paper) demonstrated that the smallest effect detectable was for sample sizes greater than *n* = 50. Our current sample size is 108, well above the minimal required sample size.

### Machine Learning Classification Based on Expert Evaluation and qEEG Features

A procedure for selecting stable biomarkers and constructing robust classification procedures has been recently published by the authors (Bosch-Bayard et al., 2018), and is briefly summarized here. To avoid capitalization upon chance, the samples are divided into a training (70%) and test set. After initial screening of variables using the independent significant features (IndFeat) (Weiss and Indurkhya, 1998) procedure, best variables for classification are selected by the elastic net regression method. This cross validated variable selection is repeated independently 1000 times and only the features significant in more than 50% of the iterations are retained for further analysis. The expected classification accuracy is evaluated by means of ROC analysis which is carried out upon a further, independent, set of 1000 cross validations. The median ROC curve is the summary operational characterization of the classifier. The distribution of the 1000 areas under the ROC curve (AUC) is used to fit a kernel probability density that allowed quantification of the variability of classification performance with variable selection. This procedure was used for:

(a) The clinical scores (standardized by the MIRT analysis) produced by each expert separately.
(b) All qEEG features (19 channels by 48 frequency bins).

Since the elastic net is a linear combination of features, its application to qEEGt (also a linear learning procedure) was not considered necessary.

## Results

### Qualitative EEG Analysis

Visual inspection of the resting EEG (AC-R and TV-A) revealed abnormalities in the EEG recordings, which were defined at the Section “Qualitative EEG Analysis”).

Visual inspection of the BNS EEG records demonstrated a preponderance of abnormalities in the PEM group. Only 6 PEM children showed the typical antero-posterior differentiation gradient expected for their age. By comparison, 26 controls showed this gradient. Additionally, the PEM group also showed a higher percentage of global abnormalities compared to controls: the total number of abnormal EEG recordings were 28 (60.9%) in PEM and 11 (17.7%) in the control group. To test if there was a significant difference between the expected and observed frequencies we employed a Chi-square test, which was significant at the *p* < 0.05 level.

To carry out more sensitive analysis of the visual inspection data, we devised Likert-type scales for the EEG based on an ordinal grading of perceived abnormality. The following five EEG items were graded: “frequency of rhythmic background activity,” “focal abnormality,” “paroxysmal activity,” “diffuse slow wave activity,” and “presence of sharp waves.” Multivariate Item Response Theory was then used to produce a global EEG abnormality score by means of a non-linear factor analysis. This analysis indicated that a single factor F1 was sufficient to describe this dataset. We came to this conclusion based on the observed Akaike information criteria (AIC) that increase when selecting two factors vs. one, indicating an undue increase of model complexity with respect to variance explained.

**FIGURE 4:**
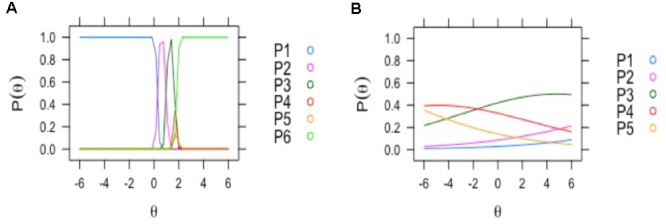
Examples of EEG visual scoring items with different assessment reliability by clinical neurophysiologists. The curves depict the probabilities of response of the two neurophysiologists to the different levels of the EEG score items (*y* axis: 0–1 probability). On the left **(A)**: discrimination profile for the item with most reliable discrimination between response alternatives (focal abnormality); all the options of classification reached the maximal probability. Right **(B)**: the profile for the item with lowest probability; “background frequency”: where the classification alternatives probabilities were near zero.

Two aspects of the MIRT factor analysis are of importance in analyzing the different EEG items (**Table 1**). The first is loading F1 of the items in the factor. The second is the reliability, of the response alternatives provided by the evaluators. Only 4 items had loadings higher than 0.7: focal abnormality, paroxysmal activity, presence of sharp waves and diffuse slow activity. These were also the items with high reliability. As can be seen from **Figure 4A** (left), the item with the best reliability, “focal abnormality,” had higher estimated probabilities for all response alternatives and did not overlap for the different levels. By contrast, the item with the lowest factor score, “background frequency” in **Figure 4B** (right), exhibited overlapping and low probabilities for the 5 levels of the item scale. This item was therefore excluded from the overall score.

**Table 1:** Item response theory analysis.

Item _j_	F1	α_j_
Focal abnormality	0.991	3.40
Paroxysmal activity	0.923	35.09
Sharp wave	0.746	2.45
Diffuse slow activity	0.704	1.68
Baseline frequency	–0.117	0.001

The loadings of the F1 components were all positive (shown in **Table 1**), except for the last item, indicating that F1 can be considered to represent the overall Neurophysiological State (NS) of the children, with higher values indicating more abnormality.

To test if there was a statistical difference of the NS score between the PEM and control groups, we used the MIRT mixed-effects procedure, as follows:

$$\begin{array}{c} \mathrm{MIRT}\mathrm{Model}1:\mathrm{Items}\sim \mathrm{mixedmirt}(\mathrm{fixed}\\ \;\;=\;\sim 1+Group,\mathrm{random}\\ \;\;\;\;\;=\;\mathrm{list}(\sim 1|\mathrm{subject},|\sim 1\mathrm{evaluator})). \end{array}$$

which combines overall score with ANOVA. The fixed effect was considered “group,” the random effects being the “evaluator” and the multiple observations for each subject. Only the difference between groups was significant (Z = -6.835, *p* < 0.0001).

### Scalp Topographic Quantitative EEG Analysis (qEEG)

The analysis of the t-test between groups using the qEEG feature set (48 bins of frequency and 19 electrodes per each subject) revealed a significant difference between the z spectra of both groups for 25 frequency/electrode combination at a level of *p* < 0.05, corrected for multiple comparisons. **Table 2** lists these features.

**Table 2:** Scalp topographic quantitative EEG analysis.

Scalp Topographic Quantitative EEG analysis (qEEG) Lead	Frequency bin (Hz)	Broad Band	Lead	Frequency bin (Hz)	Broad Band
T4	3.91	theta 1	T4	11.33	alpha 2
Pz	3.91		T4	11.72	
T4	5.07	theta 2	T5	11.72	
T4	5.86		T4	12.11	
O2	5.86		T5	12.11	
Fz	8.59	alpha 1	O1	12.11	
C3	8.59		P3	12.50	
Cz	8.98		P4	12.50	
Fp1	8.98		P4	13.67	beta 1
Fp2	8.98		T5	13.67	
F3	8.98		T4	14.45	
F4	8.98		T4	17.58	
			T4	18.36	

**FIGURE 5:**
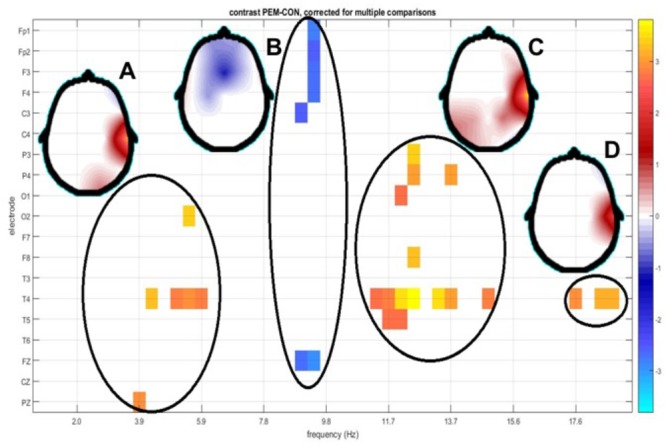
Topography of t-tests between groups scalp z spectra. The *y* axis depicts EEG derivation and the x axis shows frequency bins. Only values below the *p* < 0.05 threshold (corrected for multiple comparisons by permutation tests) are shown. Significant regions in the electrode/frequency plane are highlighted, with a circle. Regions detected were theta **(A)**, alpha1 **(B)**, alpha2 **(C)**, and beta **(D)**. Beside each circle a head plot shows the topographic distribution of the *t*-values for the most significant test in that region.

These features are grouped into four clusters that are limited to an EEG frequency band and a set of scalp electrodes. These clusters are:

1. **theta:** Increased slow activity (3.91–5.86 Hz) in electrodes T4, O2, Pz. (**Figure 5A**)
2. **alpha 1:** Decreased activity in alpha 1 (8.59–8.98 Hz) in Fp1, Fp2, Fz, F3, F4, (frontal) and C3, Cz (central) electrodes. (**Figure 5B**)
3. **alpha 2:** Increased alpha 2 (11.33–12.50 Hz) in temporal (T4, T5) parietal (P3, P4) and Occipital (O1) electrodes. (**Figure 5C**)
4. **beta:** Increased fast activity in beta 1 (13.67–18.36 Hz), more relevant in temporal (T4) and T5 and parietal P4 electrodes. (**Figure 5D**)

**FIGURE 6:**
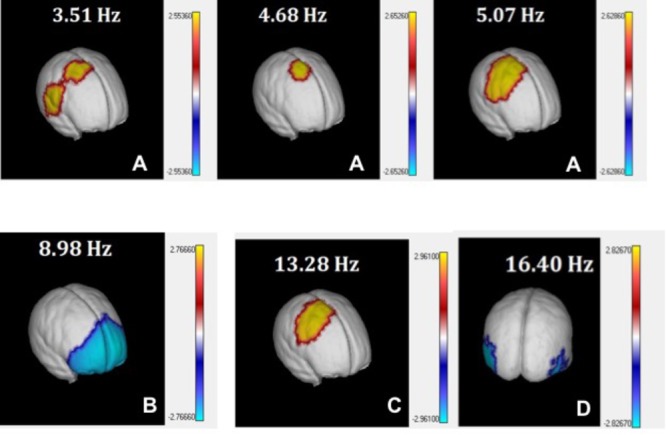
3D view of *t*-tests between PEM and Controls source z spectra. *t*-values comparing PEM and Control source spectra are plotted on the surface of the MNI average brain for selected EEG frequency bins corresponding to theta **(A)**, alpha1 **(B)**, alpha2 **(C)**, and beta **(D)**. Only values below the *p* < 0.05 threshold (corrected for multiple comparisons by permutation tests) are shown.

The schematic distribution of the electrodes significant in different frequencies is shown in **Figure 6**.

As an additional check of these results instead of analyzing the z transform of qEEG features, an ANCOVA of differences between the two groups, with group dependent age regressions was carried out. No difference between group age regressions was found and the plot of significant parameters was very similar to that of **Figure 5** and will not be discussed further.

### Quantitative EEG Tomography (qEEGt)

A similar analysis of the *t*-tests for the source z spectra identified 10 out 48 frequency bins (collapsing across all voxels) where the differences between the sources were statistically significant at *p* = 0.03, corrected for multiple comparisons. When this analysis was broken down by both voxel and frequency (see **Figure 6**), the distribution of the differences for the **PEM group** was classified in broad bands that are very similar to those from the qEEG results.

These four spectral components identified by the tomographic analysis were plotted using the AAL Atlas projected on the MNI average brain. We selected only those structures where more than 25% of voxels were activated. The relation of the anatomical structures significant for the 10 frequencies significant are shown in **Supplementary Material S3**.

The components identified were:

1. **theta:** Increase of source power at slow frequencies (3.52–5.07 Hz; *p* < 0.03) in right centro-temporo-parietal areas, especially in the supplementary motor area (SMA) of the right hemisphere. **Supplementary Table S3A** and **Figure 6A**.
2. **alpha 1:** Decrease of power in alpha at 8.98 Hz (*p* = 0.01) in widespread bilateral prefrontal areas, including the superior, medial and inferior frontal gyrus. **Supplementary Table S3B** and **Figure 6B**.
3. **alpha 2:** Increase of activity between the range of 11.33–13.28 Hz (*p* = 0.03) in centro-parietal areas of the right hemisphere. **Supplementary Table S3C** and **Figure 6C**.
4. **beta:** Decrease of activity at 16.41 Hz in bilateral occipito-temporal areas (*p* = 0.02). **Supplementary Table S3D** and **Figure 6D**.

### Machine Learning Classification Based on Expert Evaluation and qEEG Features

**FIGURE 7:**
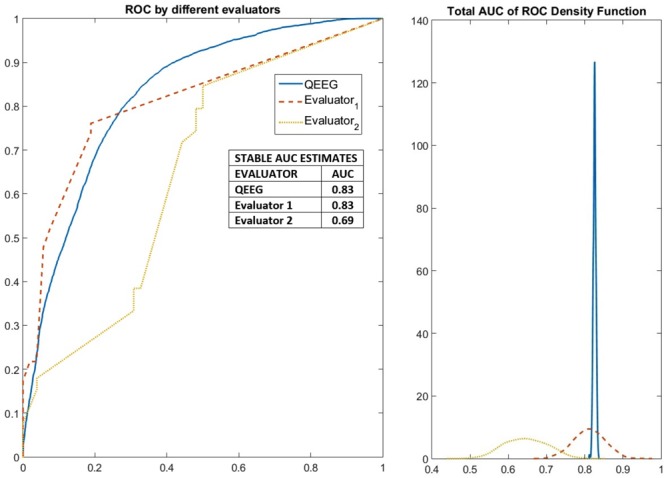
Robust measures of the performance of elastic net classification of PEM/Control groups based on features from qEEG analysis as well as from visual inspection by two EEG neurophysiologists. Results of the classification method applied to detect stable variables (selected more than 50% of the time). The performance of these stable variables in an elastic net classification of PEM/Control EEGs was evaluated in a further 1000 cross-validated fashion to yield ROC curves. On the left the median ROC curves for the two neurophysiologists and qEEG variables. On the right the probability distribution of the area under the ROC curve. Expert 1 performed above chance but poorly. Expert 2 had a variable performance with many good results. qEEG outperformed expert 1 and nearly 60% of the time expert 2.

The results of the stable machine learning classification are shown in **Figure 7** (left panel). The variability between experience evaluators is quite large, with areas under the ROC curve (AUC) ranging from 0.69 to 0.83. qEEG performs as well as the expert with higher performance. The distribution of AUC values produced by the randomization procedure (**Figure 7**-right panel) is quite informative. For all crossvalidations, AUCs are well above the chance level. However, the scores of one of the experts can range from very close to 0.5 (chance level) to about 0.7 which is a mediocre classification performance. The scores produced by the other expert is better than that of the first expert but is quite variable. The performance of qEEG is very stable and slightly above the median level of the second expert.

## Discussion

### Previous Studies of Malnutrition and EEG

The present study is unique as there are very few longitudinal EEG studies following an early episode of malnutrition. In a follow-up study of 20 South African children with histories of marasmus and 20 controls, a history of early PEM was shown to be associated with reduction in faster EEG frequencies alpha (α) rhythm for up to 12 years after a nutritional intervention (Stoch and Smythe, 1967; Baraitser and Evans, 1969). In another South African study of children who had experienced severe undernutrition (*N* = 30) (<2 SD of WHO norms) in the first year of life showed reduced alpha and higher levels of theta (slow-wave activity) relative to non-malnourished siblings (*N* = 30) and yard mates (*N* = 30) 6–12 years after recovery from malnutrition using computerized EEG (Bartel et al., 1979). In summary, previous studies using EEG in malnutrition, found reduced alpha rhythm and higher levels of theta (slow wave). These previous studies contain methodological flaws, however, including that the sample sizes reported were relatively small, lacked a control group, there were multiple stressors experienced and cofounding factors in addition to malnutrition, and insufficient control of relapses and co-morbidities.

### Analysis of Quantitative EEG Results

Comparing PEM and control children, the three first components obtained at scalp and sources analysis were similar and consistent even when the EEG at the scalp and the current densities at the sources were biophysically quite different. We now analyze each finding component in turn:

#### Excess of Theta (Slow-Wave Activity) in Pre and Postcentral Areas for Both Scalp and Source z Spectra

The excess of slow-wave activity found in the scalp z spectra of the PEM children may reflect a maturational lag in cortical development, in accordance with psychometric performance. Corning compared two groups with low and high slow EEG frequency (Corning et al., 1982) on IQ measures, and found that the group with excess of slow frequency had lower scores in verbal subtests and normal scores in non-verbal subtests. But those with the least slow frequency activity were above normal on both IQ subtests. Excess theta activity has been related to learning and attention disorders, specifically attention-deficit/hyperactivity disorder (ADHD) in qEEG studies (Clarke et al., 2002; Snyder and Hall, 2006; Barry et al., 2011). High theta levels have also been observed in children raised in aversive environments such as orphanages (Marshall et al., 2004). To our knowledge, the results for the source z spectra are the first to localize this activity at the supplementary motor area of the right hemisphere for the three theta frequencies that were statistically significant: (73.17% for 3.52 Hz, 68.29% for 4.69 Hz and 95.12% for 5.08 Hz). This could be relevant in explaining motor inhibition and language deficits in the PEM children, considering the supplementary motor area’s (SMA) crucial role in ‘self-initiated’ internal action (Nachev et al., 2008).

#### Decreased Alpha 1 Activity in Prefrontal Areas Bilaterally

The decreased alpha activity observed in the scalp z spectra of the PEM group can also be associated with a failure in functional cortical development (maturational retardation hypothesis). This hypothesis emphasizes the retardation of early developmental processes (neurulation, cellular proliferation, migration), leading to maturational arrest. Slowing of late processes, such as synaptogenesis and myelination, are more likely to result in maturational delay (Sarnat et al., 2015). It is essential to note that alpha activity in children starts 3 years after birth, almost parallel to the development of speech.

Our results are in line with those of other long-term follow-up studies in previously marasmic children showing a marked retardation in faster EEG frequencies (α rhythm) for up to 12 years following PEM and after successful nutritional treatment (Stoch and Smythe, 1967). Fundamental studies reveal that the modulations of alpha frequency among neurologically intact adults depend on the structural integrity of white matter tracts (Llinás and Steriade, 2006). Consistent with this idea, Sheridan et al. (2012) suggested that reduced alpha power observed in the EEG of children exposed to institutional rearing may be the result of delay in cortical white matter development. Results from longitudinal studies have demonstrated that normal brain development involves a linear increase in white matter from childhood to adulthood. The white matter makes the signal transduction become faster and more efficient, allowing increasingly higher frequency contributions to the overall signal. White matter maturation is an essential element of brain development and is fundamental for normal function and cognitive maturity (Luna et al., 2001; Valdés-Hernández et al., 2010). Ineffective signal transduction due to decreases in myelination caused by early malnutrition could be one explanation for the alpha power reduction in PEM. The malnutrition episode took place during the first year of life, when the most significant period of myelination occurs.

The neurophysiological impairments associated with early PEM can be permanent, often accompanied by widespread neurological disturbances involving sensory-motor activity, learning, memory, consciousness, cognition and emotion (Guedes, 2011). In the PEM group (in comparison with the control group) the source analysis related with the decrease of alpha was localized in the bilateral prefrontal region (PFC). This brain region has been implicated in planning complex cognitive behavior, personality expression, decision making, and moderating social behavior (Miller et al., 2002). The main objective of this region is to develop “*executive functions*” related to action planning, decision making, etc. According to Fuster (1988), the PFC can be subdivided into three major regions: orbital, medial, and lateral. The orbital and medial are involved in emotional behavior. The pre-frontal lateral region, which is maximally developed in the human, provides the cognitive support to the temporal behavior, speech, and reasoning.

#### Increased Alpha 2

The main difference between groups for the scalp z-spectra is the increase in fast alpha in the PEM group with a clear right asymmetry. Previous reports have indicated that this is a typical electrophysiological pattern present in depression and other emotional states (Gotlib, 1998; Debener et al., 2000; Coan and Allen, 2003, 2004; Davidson, 2004; Coan et al., 2010). Additionally, other authors have related this specific frequency rhythm with hypervigilance and anxiety in children. For a review about frontal EEG asymmetry and social behavior see (Schmidt and Miskovic, 2014).

#### Increase/Decrease of the Beta (Fast Frequencies Above 16 Hz)

The beta frequency range was the only band for which scalp z spectra and source z spectra analysis do not coincide. Scalp z spectra showed an increase of beta activity in temporal leads in PEM children, while the source z spectra for these children revealed a decrease. The scalp results are consistent with the prior literature. Scalp beta activity is believed to result from cortical/cortical and thalamo/cortical interactions. This could be an indication of immature electrical brain activity according to patterns described in normal EEG development (Matsuura et al., 1985) where a reduction of beta is expected. In fact, anterior-posterior (AP) beta decreases with age. Maturation of activity in the beta band progresses from the center to lateral and, finally, to frontal areas.

Activity in the beta frequency band is a presumed index of the level of cortical arousal. Increased beta activity has been associated with many different conditions including: externalizing spectrum disorders (Porjesz et al., 2005), ADHD, substance abuse disorders, conduct disorder, and antisocial behavior (Gilmore et al., 2010). The increased beta may thus indicate cortical hyperarousal, which is what is found in the scalp z-spectra of the PEM children.

Scalp and source results are not necessarily the same, so the discrepancy we observed is not surprising. This is a result of the way the sources are reflected on the scalp via the lead field as mentioned previously in Bosch-Bayard et al. (2001). If the electrodes are relatively close to the source, and do not receive much interference from far away sources then a high concordance between scalp and source results can be expected. This does not seem to be the case for this frequency band and will be the subject of further research.

### Relation to Cognitive and Behavioral Outcomes in the BNS

Galler and colleagues have already found deficits in intellectual performance and soft neurologic signs as well as school achievement in the PEM group versus control children of this cohort (Galler et al., 1983a,b, 1984, 1985, 1986, 1987b, 1990, 2004). These differences persisted for each outcome even after correcting for household socioeconomic factors and maternal depressive symptoms. The model employed by Galler in 1984 displayed interrelationships among previous malnutrition, soft neurological signs, classroom behavior intelligence and physical growth, demonstrating that slow motor performance was associated with lower verbal and performance IQ and the presence of attention deficit disorder, as assessed by the child’s teacher. There was a striking four-fold increase in attention problems among previously malnourished children. These problems typically did not involve hyperactivity, but rather inattention, indicative of cognitive dysregulation. Previously malnourished children also displayed more conduct problems. The inattention and conduct problems already demonstrated in the PEM group are congruent with the pattern of EEG abnormalities described above, namely the increase of theta activity in SMA, decrease of alpha in prefrontal areas, and the increase of alpha2 in SMA. A confirmation of the causal relationship between the EEG findings reported in this study and the cognitive and behavioral problems detected in these children, would be the aim of further research.

### Comparison Between Qualitative and Quantitative Analysis

The visual inspection done by the clinical neurophysiologist reported a prevalence of abnormalities (17.7%) in the BNS control group. This is much higher than what has been reported for normal children in other studies. For this reason, there might be an observer bias in this study. However, note that EEG abnormalities reported for healthy children are between 3.5 to 5% but only for epileptiform activity (Okubo et al., 1994; Riviello, 2007; Hmar et al., 2016). In our study other types of “abnormality” were included which might explain the finding better.

Nevertheless, this result underscores the need for a more objective evaluation of EEG visual findings. One approach to provide quantitative support for human evaluation is the use of a standardized clinical rating scale. These comprise a collection of items that are assigned scores which are then summed to provide an overall number. However, according to the best practices recommendation for constructing clinical evaluation scales (Hobart et al., 2007), simply summing the original ratings is to be avoided. Rather, the items should be assigned weights estimated by statistical item response theory. This was the purpose of the multivariate item response theory (MIRT) analysis we employed.

The MIRT assessment of the qualitative evaluation of the EEGs showed highly significant differences between the PEM and control groups. Visual inspection, in fact, showed that the PEM group had more diffuse slow activity, focal abnormalities, paroxysmal activity and sharp waves than the control group. There was no difference between groups for background frequency activity.

In terms of predictive value, the results shown in **Figure 7** indicate that qEEG features can provide a robust and very stable set of biomarkers with an area under the ROC curve of 0.83. This is to be contrasted with the variability between experts which ranged from 0.69 to 0.83. Thus, at worst, qEEG might substitute clinical experts. The performance might actually be better for qEEG since experts in clinical settings do not use the procedures, enhanced by MIRT, described in this paper. The Machine Learning procedure was optimized for each specialist, something that might be impractical in real life situations. In addition, qEEG is objective and free of subjective bias.

The MIRT results are noteworthy in light of the strengths and limitations of qEEG and qEEGt which are based on spectral analysis of the EEG. Spectral analysis is optimal in identifying the frequency content of signals, even picking up on small amplitude oscillations that are missed by visual inspection. It is interesting to speculate that the diffuse slow wave activity is well captured by spectral features. On the other hand, visual scoring of the base frequency of the EEG only reflects the most dominant frequencies of the EEG and the analysis of other frequencies is an area in which qEEG may outperform visual inspection. Note that MIRT eliminated background frequency as an interesting variable which is the main characteristics quantified by qEEG.

However, it is also instructive to analyze the features that are not currently analyzed by qEEG but were revealed to have additional descriptive power in visual scoring of EEG. These were paroxysmal activity, focal abnormalities and sharp waves, are transient phenomena, that are missed by spectral analysis, which is a tool for stationary signals. A qEEG method based on non-linear and non-stationary features of the EEG was proposed by Valdes et al. (1999). This alternative feature set must be studied with the expectation that it might provide increased qEEG diagnostic accuracy beyond that described in this paper. Currently we use qEEG and visual inspection separately to analyze EEG, but combining these methods may have utility for automatic diagnosis.

### Future Work

Future work will address several limitations of our current approach:

(1) EEG and other neuroimaging studies of the two cohort groups are being undertaken in the same individuals 40 years later to evaluate the evolution and permanence of the early life neural fingerprints of PEM over the life span.
(2) Both linear (spectral) and non-linear features must be considered for the current EEG dataset as well as for future EEG recordings to increase predictive power.
(3) In future studies, EEG source methods will be improved with the use of individualized head models from MRI data (not available in the 1970’s).
(4) Features obtained from source connectivity measures will also be included.
(5) The relationship between these EEG findings and the cognitive and behavioral function of the population will be further studied.
(6) Most importantly, the EEG feature sets will be integrated within a stochastic disease progression model that will also include demographic, cognitive, behavioral, physiological and epigenetic variables.

The objective of this and ongoing studies is not only to understand better the long-term effects of early PEM, but also to predict response to treatment as the goal for a health impact of this research.

All data and programs for used in this study will be available via the open source portal CBRAIN^3^ (Sherif et al., 2014)

## Conclusion

To our knowledge, this is the first study to identify quantitative EEG (qEEG) in individuals who suffered from PEM during the first year of life. In spite of the attrition due to loss of early records, the final group is much larger than any study of EEG-PEM to date. In addition, this is the only report on EEG and childhood malnutrition that studies EEG sources by means of tomographic qEEG.

Significant differences between both groups in the z spectra (for all locations and frequencies) were found in the following four clusters

(A) Increased theta activity (3.91–5.86 Hz) in electrodes T4, O2, Pz and in the sources of the supplementary motor area (SMA); (B)
(B) Decreased alpha1 (8.59–8.98 Hz) in Fp1, Fp2, Fz, F3, F4, C3, Cz electrodes and sources of widespread bilateral prefrontal areas.
(C) Increased alpha2 (11.33–12.50 Hz) in T4, T5, P3, P4, O1 electrodes as well as in sources in central-parietal areas of the right hemisphere.
(D) Increased beta (13.67–18.36 Hz), in T4, T5, and P4 electrodes and decreased in the sources of bilateral occipital-temporal areas.

In addition, Multivariate Item Response Theory (MIRT) of EEG expert visual inspection revealed a neurophysiological latent variable which indicated excessive paroxysmal and focal abnormality activity in the PEM group at the (*p* < 0.0001) level.

Machine learning performance using the elastic net classifier shows that discrimination between index and the control groups is very stable when based on qEEG features, with the median value higher than that of classifiers based on scores by the best expert.

These findings suggest that both, the quantitative (qEEG) and the tomographic (qEEGt) might may be a source of scalable and affordable biomarkers for assessing the long term brain impact of protein energy malnutrition (PEM).

## Author Contributions

PV-S directed this collaboration, designed the research plan, and originated the signal processing and statistical analyses used. JG is the co-founder of the Barbados Nutrition Study, and designed and directed this 45+ year study. CB has served as the field director of the BNS since 2005. JG, LP, and RI were involved with original EEG recording and recovery of the archival data. AT-C was responsible for pre-processing the EEG data. JB-B was responsible for qEEG and qEEGt analysis. LG-G oversaw statistical analysis together with AT-C, MB-V, and YG. MB-V was responsible for quality control of the original EEG data together with AC-R and TV-A. AT-C, JB-B, and MB-V carried out the initial manuscript preparation(with equal contribution). PV-S, MB-V, JG, and AR conducted the literature review and final manuscript preparation.

## Conflict of Interest Statement

The authors declare that the research was conducted in the absence of any commercial or financial relationships that could be construed as a potential conflict of interest.

## Footnotes

1 This paper is dedicated to the memories of Sir Dr. Frank C. Ramsey and E. Roy John. Ramsey was the former Director of the Nutrition Centre of Barbados, who, with Prof. Galler, established the Barbados Nutrition Study, followed the BNS children for most of their lives and contributed to the elimination of malnutrition from Barbados. John created the Brain Research Laboratories at NYU, was a pioneer in quantitative EEG analysis and worked closely with the Cuban Neuroscience Center.

2 www.ifcn.info/

3 https://mcin-cnim.ca/technology/cbrain/

## Supplementary Material

FIGURE S1 The original (G1 first generation) cohort evaluated in 1978 consisted of 258 children, and 137 of these EEG recordings were recovered in 2016.
