# Supplementary figures and images for: Quantitative EEG Tomography of Early Childhood Malnutrition
Source: Front Neurosci. 2018 Aug 28;12:595. doi: 10.3389/fnins.2018.00595 (PMC6127649; doi:10.3389/fnins.2018.00595)

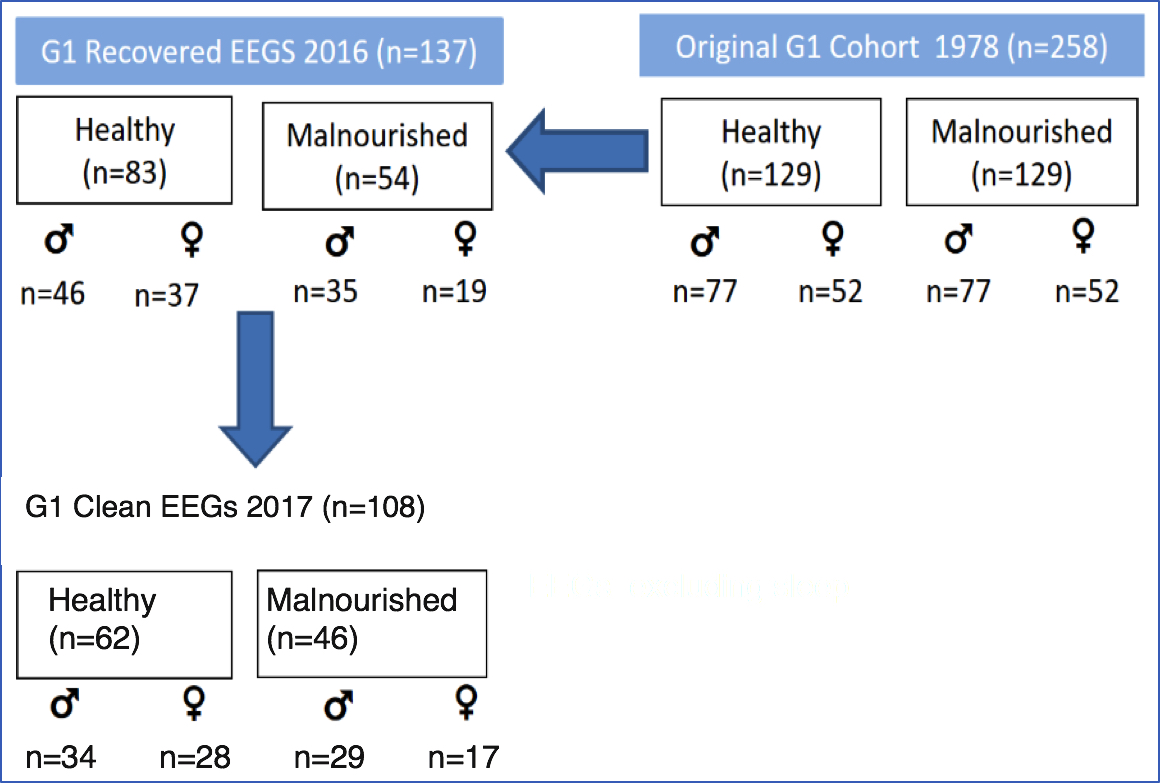

Supplement: FIGURE S1 — The original (G1 first generation) cohort evaluated in 1978 consisted of 258 children, and 137 of these EEG recordings were recovered in 2016. [file Image_1.JPEG]
