# Supplementary material for: Quantitative EEG Tomography of Early Childhood Malnutrition
Source: Front Neurosci. 2018 Aug 28;12:595. doi: 10.3389/fnins.2018.00595 (PMC6127649; doi:10.3389/fnins.2018.00595)
Supplement: Supplementary file 2 [file Table_1.DOCX]

# Supplementary material 1

## Modified GTE scale.

1. **Frequency of rhythmic background activity**. This the predominant EEG activity. This is usually but not necessarily synonymous of alpha activity, depending of the conditions of the recordings.

0= >8.1 Hz

1= 7.1-8 Hz

2= 6-7 Hz

1. **Diffuse Slow activity.** The presence of persistent non‑rhythmic theta‑delta slow waves localized in broader regions.

0= None

1= Slow theta

2= intermittent theta + sporadic delta

3= intermittent theta + intermittent delta

1. **Paroxysmal activity**. This is related to the activity with sudden rapid onset, rapid attainment of a maximum, and abrupt termination; distinguished from background activity, such as spikes, and spike and wave. The spikes have the duration by convention, between 20 and 70 msec. Spike and wave complex is when one spike is followed by a delta frequency wave.

0= None

1= Paroxysmal slow activity

2= Spikes

3= Spikes + waves item

1. **Focal abnormality**. Localization of the EEG abnormalities.

0= No focal abnormality

1= Slight unilateral abnormality

2= Slight bilateral abnormality

3= Severe unilateral + Slight contralateral

4= Severe bilateral

5= Multifocal

1. **Sharp wave activity**. Paroxysmal activity that lasts from 70‑200 msec.

0= None

1= Sporadic sharp waves

2= Frequent Sharp waves

1. **GTE score**= (sum 1-5)+1
