# Supplementary material for: Quantitative EEG Tomography of Early Childhood Malnutrition
Source: Front Neurosci. 2018 Aug 28;12:595. doi: 10.3389/fnins.2018.00595 (PMC6127649; doi:10.3389/fnins.2018.00595)
Supplement: Supplementary file 3 [file Table_2.DOCX]

Supplementary Material 3.

Description of the structural areas with more than the 25 % of voxels statistically significant in the source analysis

| **Table 3.A Structures with statistical significance for Theta** | | | |
| --- | --- | --- | --- |
| **Structure AAL atlas name** | **Frequencies** | | |
|  | **3.52 Hz** | **4.69 Hz** | **5.08 Hz** |
| Precentral_R | 42 |  | 94 |
| Frontal_Sup_R | 27 | 25 | 38 |
| Frontal_Mid_R | 16 | 4 | 40 |
| Frontal_Inf_Oper_R |  |  | 39 |
| Supp_Motor_Area_R | 73 | 68 | 95 |
| Insula_R | 32 |  |  |
| Cingulum_Mid_R | 44 | 3 | 56 |
| Postcentral_R | 27 |  |  |
| SupraMarginal_R | 29 |  |  |
| Temporal_Sup_R | 55 |  |  |
| Calcarine_L |  |  | 24 |
| Calcarine_R |  |  | 45 |
| Lingual_L |  |  | 63 |
| Lingual_R |  |  | 65 |
| Postcentral_R |  |  | 27 |

| **Table 3.B Structures with statistical significance for alpha 1** | |
| --- | --- |
| **Structures AAL atlas** | **Frequency** |
|  | **8.98 Hz** |
| Frontal_Sup_L | 47 |
| Frontal_Sup_R | 37 |
| Frontal_Mid_L | 39 |
| Frontal_Mid_R | 28 |
| Frontal_Inf_Tri_L | 37 |
| Frontal_Inf_Tri_R | 28 |
| Frontal_Inf_Orb_L | 38 |
| Frontal_Inf_Orb_R | 54 |
| Frontal_Sup_Medial_L | 62 |
| Frontal_Sup_Medial_R | 73 |

| **Table 3.C Structures with statistical significance for alpha 2** | | | | | |
| --- | --- | --- | --- | --- | --- |
| **Structures AAL atlas** | **Frequencies** | | | | |
|  | **11.33** | **11.72** | **12.11** | **12.89** | **13.28** |
| Precentral_L |  |  | 100 |  |  |
| Precentral_R |  | 100 | 100 |  | 63 |
| Frontal_Sup_L | 11 |  | 47 |  |  |
| Frontal_Sup_R | 45 | 48 | 56 | 7 | 38 |
| Frontal_Mid_L |  |  | 52 |  |  |
| Frontal_Mid_R | 41 | 63 | 67 | 4 | 37 |
| Frontal_Inf_Oper_R | 7 | 100 | 100 |  | 36 |
| Frontal_Inf_Tri_R | 2 | 55 | 75 |  | 3 |
| Supp_Motor_Area_L | 7 | 29 | 100 |  |  |
| Supp_Motor_Area_R | 95 | 100 | 100 | 20 | 93 |
| Frontal_Sup_Medial_L | 3 | 3 | 26 |  |  |
| Frontal_Sup_Medial_R | 12 | 18 | 27 |  |  |
| Insula_L |  |  | 32 |  |  |
| Insula_R |  | 47 | 91 |  |  |
| Cingulum_Mid_L | 13 | 40 | 100 |  |  |
| Cingulum_Mid_R |  | 66 | 97 | 19 | 44 |

| **Table 3.D Structures with statistical significance for beta** | |
| --- | --- |
| **Structure AAL atlas** | **Frequency 16.41 Hz** |
| Lingual_L | 48 |
| Occipital_Mid_L | 25 |
| Fusiform_L | 69 |
| Fusiform_R | 36 |
| Temporal_Mid_L | 41 |
| Temporal_Inf_L | 62 |
| Temporal_Inf_R | 29 |
